# Internal Load and Technical-Tactical Characteristics in Small-Sided Games: An Investigation in Adolescent Water Polo Players

**DOI:** 10.3390/sports14060249

**Published:** 2026-06-17

**Authors:** Andrea Perazzetti, Federico Carrozza, Francesca Martusciello, Milivoj Dopsaj, Daniele Ruffelli, Antonio Tessitore

**Affiliations:** 1 Degree Course in Sciences of Motor Activities, Sports and Psychomotor Education, Faculty of Law, Telematic University Giustino Fortunato, 82100 Benevento, Italy; 2 SS Lazio Nuoto, 00145 Rome, Italy; federico.carrozza@libero.it (F.C.); turpe@hotmail.it (D.R.); 3 Department of Movement, Human and Health Sciences, University of Rome “Foro Italico”, 00135 Rome, Italy; francesca.martusciello@uniroma4.it (F.M.); antonio.tessitore@uniroma4.it (A.T.); 4 Faculty of Sport and Physical Education, University of Belgrade, 11000 Belgrade, Serbia; milivoj.dopsaj@fsfv.bg.ac.rs

**Keywords:** small-sided games, performance analysis, TSAP, RPE, enjoyment, youth athletes

## Abstract

The aim of this study was to analyze the effects of different small-sided game (SSG) formats on internal load, perceived enjoyment, and technical-tactical performance in elite youth water polo players. Twenty male athletes (U16, n = 10; U18, n = 10) performed in three 4 vs. 4 SSG formats with different time of ball possessions and size of field areas. Technical-tactical variables were assessed using the Team Sport Assessment Procedure (TSAP), while internal load and enjoyment were measured through session-RPE (s-RPE) and a 7-point enjoyment Likert scale (ENJ). Data were analyzed using linear mixed-effects models and Spearman correlations. SSG format significantly influenced internal load, with higher RPE values (F = 6.878; *p* = 0.004) and s-RPE (F = 6.27; *p* = 0.006) observed in larger formats of the SSG. Technical-tactical indices were also affected, with significant differences found for volume of play (VP) (F = 17.041; *p* < 0.001) and performance score (PS) (F = 18.574; *p* < 0.001), showing higher values in the smallest format (SSG1). Enjoyment differed between categories (F = 13.136; *p* = 0.003), with higher scores in U16 players. No significant correlations were found between final RPE and TSAP indices (*p* > 0.05). These findings suggest that SSGs are effective tools for simultaneously developing physical and technical-tactical skills. Coaches should manipulate task constraints to balance training intensity and skill development, while also enhancing player motivation and engagement.

## 1. Introduction

Water polo is a highly demanding water-based contact team sport that requires players to engage in intense bursts of sprint swimming while constantly participating in technical and tactical activities [1,2]. The high-intensity nature of play, with approximately half of the total distance covered at speeds > 1.4 m·s^−1^ [3], require that players perform movements with frequent changes of speed and direction, interspersed with incomplete recovery periods [4]. Thus, players’ levels of aerobic, anaerobic, and strength conditioning are crucial for their in-game performance, with consistent differences observed between national and international levels [5]. Although the overall physiological load appears comparable among players in different positions, the different technical and tactical tasks they perform, along with the more dynamic body contacts experienced by centers (defenders and forwards), as well as the higher number of swimming bouts undertaken by peripheral players, help explain the trend of higher s-RPE values observed in the latter [6,7]. Consequently, considering the continuous technical, tactical, and conditioning evolution of play, along with the new rules introduced in 2025, there is a need for new training approaches that integrate physical and technical-tactical aspects, closely aligned with the established technical and tactical model, to better reflect real game demands [8].

The use of training methods that “differ from standard front-crawl swimming, incorporating larger muscle mass, physical contact, and ball skills” has been fostered for a long time in water polo [9]. This approach is gradually evolving, with coaches and strength and conditioning specialists increasingly proposing more integrated training methods. However, there are also coaching staff continuing to use a siloed approach, keeping physical and technical-tactical training separate with a low level of integration, a strategy also depending on the coaching philosophy of the different national water polo coaching schools [10]. On the other hand, specific anaerobic endurance conditioning techniques, such as sprint swimming and in-water jumping, have been correlated with offensive and defensive playing situations. Therefore, these techniques should be specifically developed to mimic true game constraints, such as playing area and player positions [11]. Botonis et al. [12] used a repeated-measures design to compare the physiological effects on elite water polo players of high-intensity swimming with versus without a ball. The findings of this study showed that both high-intensity swimming and water polo-specific counterattack drills were effective for conditioning, with the latter offering additional tactical benefits. Furthermore, the reduced blood lactate concentrations and lower perceived exertion observed during ball-based training may result from players engaging in low-intensity activity when positioned on the ‘weak side.’ These periods of reduced demand, occurring when the ball is distant, likely facilitate lactate clearance.

Studying the effects of situational variables on offensive performance indicators in elite water polo, García-Ordóñez et al. [13] emphasize the fact that coaches should use identified variables to implement training simulations that mimic competition scenarios. In this regard, game-based training has been identified as an effective method for conditioning team-sport athletes by integrating both physical and technical-tactical components, often matching or even exceeding the fitness gains of traditional training methods [14]. These exercises are known as small-sided games (SSGs) and consist of modified game formats where key constraints (e.g., number of players, pitch size, duration, coach encouragement, etc.) can be adjusted to simulate the specific movement patterns and physiological demands of the game [15]. These characteristics make SSG a flexible and effective tool for simultaneously developing physical and technical-tactical aspects, allowing coaches to better align training with the specific demands of the game. Additionally, beyond enhancing physical performance, SSGs promote greater cognitive engagement while sharpening technical skills and decision-making skills under time constraints [16].

Considering the current understanding of SSGs in team sports, their application in water polo still warrants further investigation, particularly regarding how different task constraints influence workloads, as well as the medium- and long-term effects of SSG-based training on physiological adaptations and tactical behavior [17]. For this reason, the present study provides scientific insights into the effectiveness of three SSG formats for elite youth water polo players, based on different playing areas.

Specifically, we hypothesized that different SSG formats would elicit different internal load, enjoyment, and technical-tactical responses, and that these responses would differ between the two age categories of adolescent water polo players (U16 and U18).

## 2. Materials and Methods

### 2.1. Experimental Design

This study analyzed technical-tactical indices and monitored internal load and enjoyment in a group of 20 elite male youth water polo players, divided into two age categories (U16, n = 10; U18, n = 10), during play in three different formats (i.e., different pitch sizes) of a 4v4 small-sided game.

**Figure 1:**
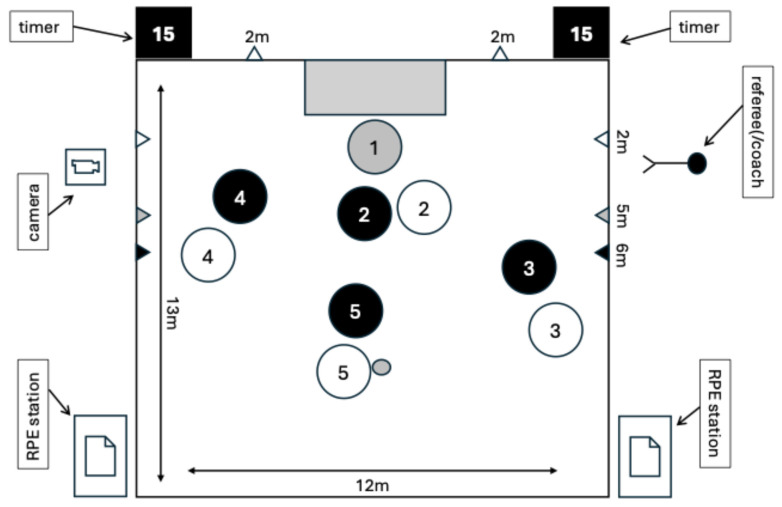
SSG1 format (13 × 12 m).

**Figure 2:**
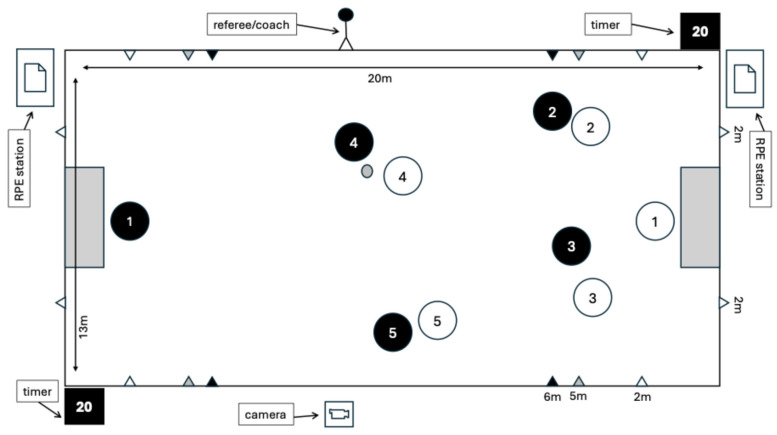
SSG2 format (20 × 13 m).

**Figure 3:**
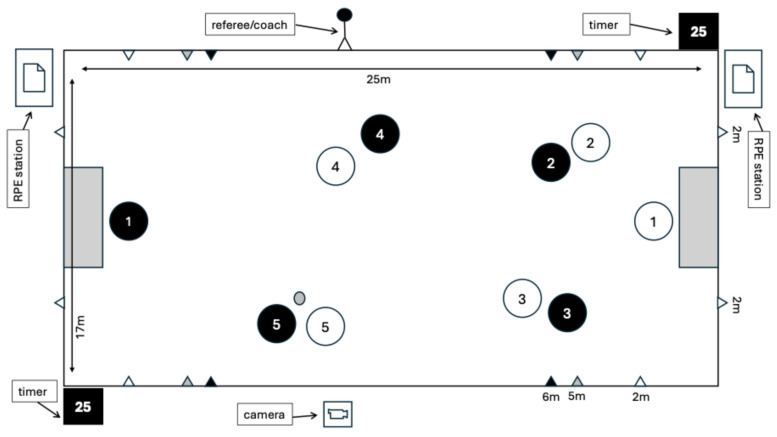
SSG3 format (25 × 17 m).

The number of players in the SSG (n = 8, 4 vs. 4) remained constant across all formats, with no substitutions, except for the goalkeeper position. In the first format, only one goalkeeper was used, while in the other two formats both goalkeepers were involved. Players maintained the same teams across all three formats, wearing the same colors (white vs. black) and cap numbers (numbers 2 to 5). The field dimensions and ball possession time for each SSG were as follows (goalkeepers were excluded from the calculation of playing area in terms of square meters per field player):

- SSG1: 13 × 12 m, 19.5 square meters per each field player, 15 s for the first possession and 10 s for the second possession (one goal, one goalkeeper, and 8 field players) (Figure 1);
- SSG2: 20 × 13 m, 32.5 square meters per each field player, 20 s for the first possession and 12 s for the second possession (two goals, two goalkeepers, and 8 field players) (Figure 2);
- SSG3: 25 × 17 m, 53.1 square meters per each field player, 25 s for the first possession and 15 s for the second possession (two goals, two goalkeepers, and 8 field players) (Figure 3).

The study was conducted during the 2024/25 competitive season, in the month of June. Specifically, for both age categories, the SSG sessions were carried out over three consecutive Mondays, each preceded by a rest day on Sunday.

The game formats used in the study were selected because they were commonly applied by the coach during regular training sessions. Therefore, the athletes were already familiar with these SSGs, which were also regularly performed outside the context of this research. Nevertheless, before each session, the rules and game formats were explained again by the coach to ensure consistency and clarity.

The basic rules were based on traditional water polo, with some modifications: (i) each SSG consisted of three periods of 6 min of continuous play, interspersed with 1 min and 30 s of passive recovery; (ii) exclusions did not result in a full numerical superiority situation (i.e., for the entire duration of the second possession), as the excluded player re-entered the field immediately after reaching the exclusion box area; (iii) players were not allowed to remain in front of the goal for more than half of the first ball possession; (iv) in the SSG1 format, after each change of possession, the attacking team had to bring the ball back beyond the 6 m line, allowing the opponents to reorganize in defense.

Each SSG was preceded by a standardized 15 min warm-up. All SSGs were recorded using a camera (Sony FDR-AX43; Sony, Tokyo, Japan) mounted on a tripod, positioned in the central area of the pool on the opposite side of the referee (coach), to ensure a stable and clear view.

Subsequently, notational analysis data were collected using an adapted version of the observational tool the ‘Team Sport Assessment Procedure’ (TSAP) [18], already validated in previous water polo studies [19] (Table 1). Received balls (RBs) and conquered balls (CBs) were considered as variables related to ball possession acquisition, while neutral balls (NBs), lost balls (LBs), offensive balls (OBs), and successful shots (SSs) were considered as variables related to ball possession management. As performance indicators, the following were calculated: game volume (VP = RB + CB), efficiency index (EI = (OB + SS)/(10 + LB)), and performance score (PS = (VP/2) + (EI × 10)), which were derived from TSAP variables (Table 1).

At the end of each game period, players were asked to individually report their perceived exertion (RPE) using the question: “Indicate the intensity level of perceived effort during the current training session.” After completing the SSG, players continued the training with a free shooting session for the remaining time (approximately 30 min).

At the end of the training session, players were again asked to report their final RPE for the entire session and their perceived level of enjoyment using the question: “Indicate the level of enjoyment perceived during the current training session.”

For RPE, the Italian version of the Borg category-ratio 10 scale (CR-10), modified by Foster et al. [20], was used. Internal load for each player was calculated using the session-RPE (s-RPE) method for each game period and for the entire SSG session.

At the same time, perceived enjoyment was assessed using the Italian translation and adaptation of the Exercise Enjoyment Scale [10,21]. To minimize individual variability in the perception of effort and enjoyment, players were asked not to use specific recovery strategies and to maintain similar lifestyle habits and nutritional behaviors to their teammates throughout the duration of the study (3 weeks).

### 2.2. Participants

The study involved a total of 20 male players, divided into Under 16 (n = 10) and Under 18 (n = 10) categories, including 16 field players (all perimeter players) and 4 goalkeepers (two per category). Goalkeepers were excluded from the statistical analyses, as they were essential for the game but not relevant for TSAP analysis and perceptual scales due to their specific performance profile [22].

All players belonged to the S.S. Lazio Nuoto Italian club and were part of elite youth teams. One month after the current study, both categories (U16 and U18) won the Italian national championship titles in their respective final tournaments in July 2025. In addition, five U18 players competed at the U18 Men’s European Championships, while 1 player competed at the U16 Men’s World Championships.

In general, U16 players performed five training sessions per week plus two gym sessions, for a total of approximately 9.5 h per week. In contrast, U18 players completed seven training sessions per week plus three gym sessions, for a total of approximately 13.5 h per week.

Approval for data collection was obtained from the University Research Ethics Committee (CAR-IRB) of the University of Rome “Foro Italico”, which confirmed that the study complied with ethical principles and Good Clinical Practice guidelines (GCP-ICH) and assigned the protocol number CAR 99/2021.

Informed consent was obtained from the players’ parents (or legal guardians) after they were informed about the main objectives of the study.

### 2.3. Procedures

Players were familiarized with the RPE and Enjoyment scales during the two weeks preceding the start of the study. During this period, they received printed copies of the scales with instructions on how to interpret them during and after each SSG. The tests were conducted in a 33 × 20 m swimming pool. The playing area was carefully measured using a 30 m measuring tape (Stanley, Italy), and cones were used to mark the 2, 5, and 6 m lines, the goal area, and the exclusion area.

Ball possession time was monitored using digital shot clocks (Favero Electronics, Italy). Matches were recorded using a camera (Sony FDR-AX43; Sony, Tokyo, Japan) mounted on a tripod and positioned at the center of the pool, and were subsequently analyzed manually using Microsoft Excel 2021 MSO (Version 2507 Build 16.0.19029.20136).

During the data collection period, for each SSG, RPE values were recorded at the end of each period (1st, 2nd, and 3rd) and at the end of the entire session, while Enjoyment was assessed only at the end of the session, within approximately 30 min after the completion of the training. Before each session, players placed their individual sheets with the perception scales on the poolside, maintaining adequate distance to avoid influencing each other.

**Figure 4:**
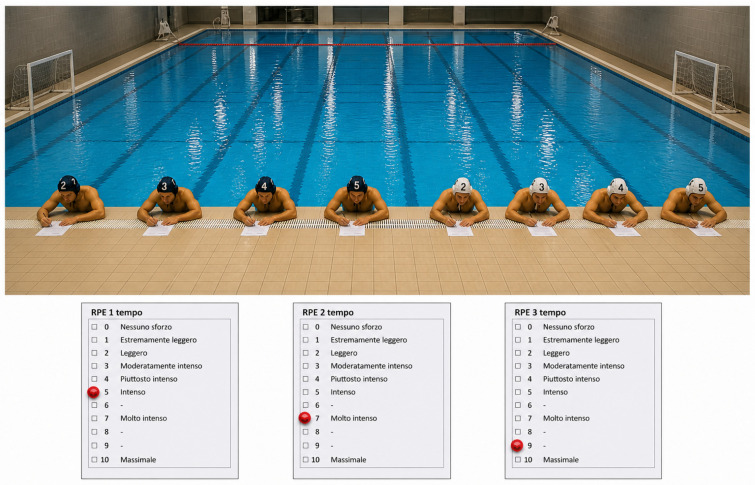
Example of RPE scale administration during the 1st, 2nd and 3rd game periods, respectively (Illustrative image generated using artificial intelligence, ChatGPT, OpenAI, based on GPT-5.5).

At the end of each period, during the 1 min 30 s recovery, and at the end of the training session, players marked their perceived effort and enjoyment by placing a pin on the corresponding point of the scales (Figure 4).

Participants reported their perceived exertion (RPE) on a CR-10 scale (0 = “No effort”, 10 = “Maximal”) for each game period (6 min) and within approximately 30 min after the end of the training session. Players also reported their perceived enjoyment using a 7-point Likert scale (0 = “No enjoyment”, 7 = “Extremely enjoyable”) within 30 min after the end of the training session.

Each SSG was recorded using a video camera and subsequently analyzed manually in Microsoft Excel 2021 MSO (Version 2507 Build 16.0.19029.20136), using the TSAP tool to record technical-tactical variables for each player.

### 2.4. Statistical Analysis

Descriptive statistics for all variables were reported as mean ± standard deviation. Normality of the distributions was assessed using the Shapiro–Wilk test. Variables were analyzed using non-parametric tests since they did not meet normality assumptions.

To evaluate the effect of contextual factors on internal load (RPE), perceived enjoyment (ENJ), and TSAP technical-tactical indices (RB, CB, VP, NB, LB, OB, SS, EI, and PS), Linear Mixed-Effects Models were applied. Specifically, the following fixed factors were included: (i) player category (U16 vs. U18), and (ii) type of small-sided game (SSG) (SSG1, SSG2, and SSG3). Players were included as a random effect to account for repeated measures on the same subjects.

For each significant main effect, effect size (Cohen’s d) was calculated and interpreted as follows [23]: 0.2 trivial; 0.2–0.6 small; 0.6–1.2 moderate; 1.2–2.0 large; >2.0 very large. All results related to main effects were reported as F and *p* values. In addition, 95% confidence intervals (95% CI) for estimated marginal means were calculated and reported to improve interpretability and provide a measure of the precision of the observed effects.

Finally, to explore the relationships between internal load, perceived enjoyment, and technical-tactical indices, Spearman’s correlation was calculated to identify potential significant associations between variables.

All analyses were performed using Microsoft Excel 2021 MSO (Version 2507 Build 16.0.19029.20136) and Jamovi (version 2.6.44, x64; Jamovi, Sydney, Australia). The level of statistical significance was set at *p* < 0.05.

## 3. Results

Table 2 shows the final RPE and Enjoyment values, divided by category and SSG format.

**Figure 5:**
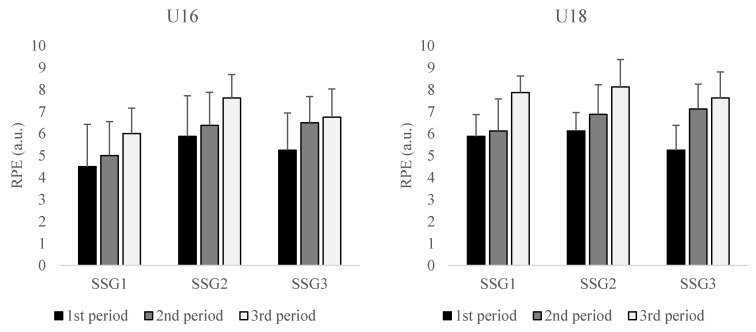
Mean ± SD of RPE values across game periods for the different matches in the U16 (**left**) and U18 (**right**) categories. Note. RPE—rate of perceived exertion; SSG—small-sided game.

Figure 5 shows the RPE values across the three game periods for the different SSG formats in the U16 and U18 teams.

**Figure 6:**
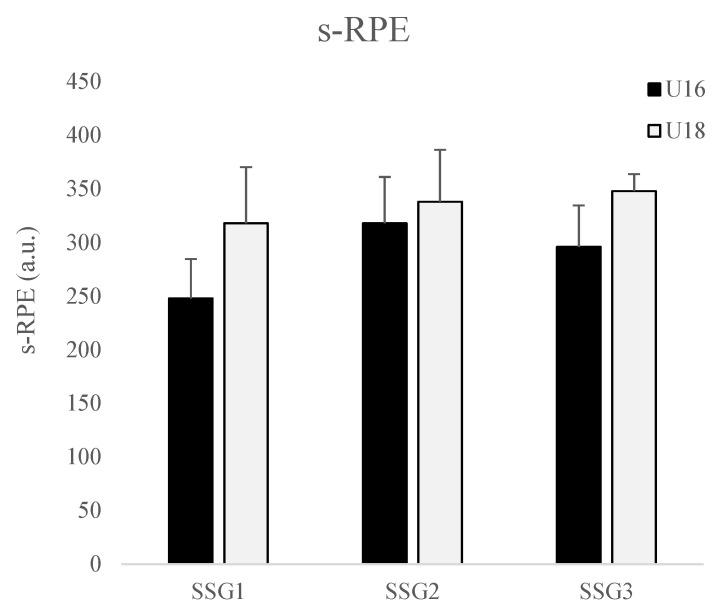
Mean ± SD of s-RPE values across the different SSG formats for both categories. Note. s-RPE—session rated perceived exertion method; SSG—small-sided game.

Figure 6 shows the s-RPE values across the different SSG formats for the U16 and U18 teams.

**Figure 7:**
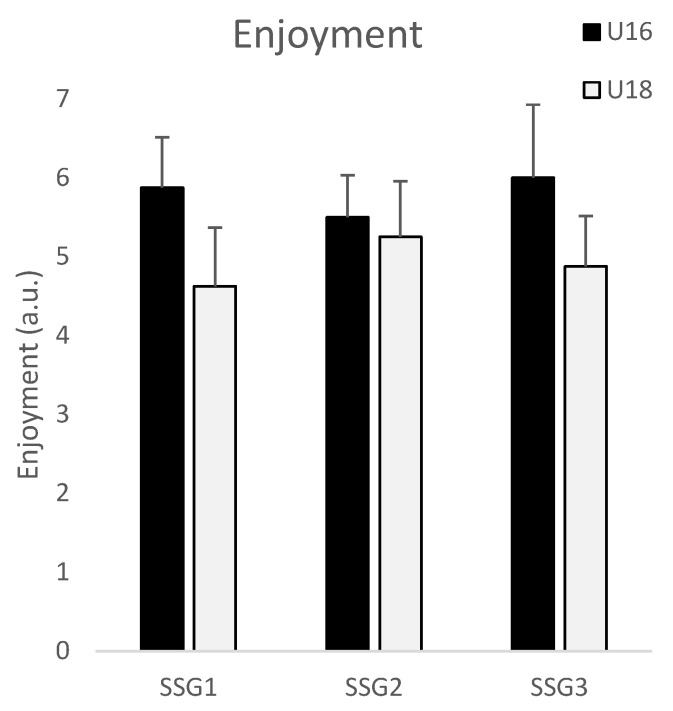
Mean ± SD of Enjoyment values across the different SSG formats for both categories. Note. SSG—small-sided game.

Figure 7 shows the Enjoyment values across the different SSG formats for the U16 and U18 teams.

Table 3 shows the total number of events for TSAP variables across the different SSG matches, divided by U16 and U18 categories.

Table 4 shows the descriptive analysis of TSAP variables and indices for both categories across the different SSG formats.

Table 5 shows the correlation analysis between RPE and TSAP variables and indices across the different SSG formats.

### 3.1. RPE Values

The linear mixed-effects model analysis showed that the format of SSG significantly influenced RPE values in the 1st period (F = 3.85; *p* = 0.033). Specifically, the post hoc test (Bonferroni) revealed a significant difference between SSG1 and SSG3 (estimate = 0.938; mean difference ± SE = −0.938 ± 0.37; 95% CI = 0.2, 1.65; Cohen’s d = 0.63, moderate).

The RPE in the 2nd period varied based on the type of SSG (F = 6.805; *p* = 0.004). The post hoc test (Bonferroni) revealed a significant difference between SSG1 and SSG2 (estimate = 1.063; mean difference ± SE = −1.063 ± 0.365; 95% CI = 0.346, 1.779; Cohen’s d = 0.73, moderate) and between SSG1 and SSG3 (estimate = 1.25; mean difference ± SE = −1.25 ± 0.365; 95% CI = 0.534, 1.966; Cohen’s d = 0.93, moderate). In the 3rd period, the RPE was also significantly influenced by the type of SSG (F = 4.57; *p* = 0.019). The post hoc test (Bonferroni) revealed a significant difference between SSG1 and SSG2 (estimate = 0.938; mean difference ± SE = −0.938 ± 0.321; 95% CI = 0.308, 1.567; Cohen’s d = 0.65, moderate). The type of SSG significantly influenced final RPE values, as shown by the linear mixed-effects model (F = 6.878; *p* = 0.004). The post hoc test (Bonferroni) revealed a significant difference between SSG1 and SSG2 (estimate = 0.938; mean difference ± SE = −0.938 ± 0.292; 95% CI = 0.365, 1.51; Cohen’s d = 0.73, moderate) and between SSG1 and SSG3 (estimate = 0.938; mean difference ± SE = −0.938 ± 0.292; 95% CI = 0.365, 1.51; Cohen’s d = 0.78, moderate).

The Spearman’s correlation showed that RPE in the first period was positively correlated with RPE in the second period (rho = 0.594; *p* < 0.001) and with the third period (rho = 0.44; *p* = 0.002). RPE in the second period showed an even stronger positive correlation with the third period (rho = 0.685; *p* < 0.001), while regarding mean RPE between the three periods and final RPE at the end of session, Spearman’s correlation analysis showed a significant association (rho = 0.716; *p* < 0.001).

### 3.2. Enjoyment Values

The linear mixed-effects model analysis showed that age category significantly influenced final Enjoyment values (F = 13.136; *p* = 0.003). The post hoc test (Bonferroni) revealed a significant difference between U16 and U18 (estimate = −0.875; mean difference ± SE = −0.875 ± 0.241; 95% CI = −1.348, −0.402; Cohen’s d = 1.21, large).

The Spearman’s correlation between final RPE and final Enjoyment did not show a statistically significant association (rho = −0.129; *p* = 0.382).

### 3.3. s-RPE Values

The study showed that the type of SSG significantly influenced s-RPE values (F = 6.27; *p* = 0.006). The post hoc test (Bonferroni) revealed a significant difference between SSG1 and SSG2 (estimate = 16.88; mean difference ± SE = −16.88 ± 5.18; 95% CI = 6.73, 27.02; Cohen’s d = 0.91, moderate) and between SSG1 and SSG3 (estimate = 14.63; mean difference ± SE = −14.63 ± 5.18; 95% CI = 4.48, 24.77; Cohen’s d = 0.81, moderate).

### 3.4. TSAP Values

Results showed that the type of SSG significantly influenced RB values in the TSAP (F = 7.871; *p* = 0.002). The post hoc test (Bonferroni) revealed a significant difference between SSG1 and SSG3 (estimate = −6.188; mean difference ± SE = 6.19 ± 1.56; 95% CI = −9.25, −3.122; Cohen’s d = 1.37, large). CB values in the TSAP were also significantly influenced by the type of SSG (F = 8.788; *p* < 0.001). The post hoc test (Bonferroni) revealed a significant difference between SSG1 and SSG2 (estimate = −2.375; mean difference ± SE = 2.375 ± 0.616; 95% CI = −3.582, −1.168; Cohen’s d = 1.19, moderate) and between SSG1 and SSG3 (estimate = −2.063; mean difference ± SE = 2.063 ± 0.616; 95% CI = −3.269, −0.856; Cohen’s d = 0.93, moderate).

The VP in the TSAP was significantly influenced by the type of SSG (F = 17.041; *p* < 0.001). Specifically, the post hoc analysis (Bonferroni) revealed a significant difference between SSG1 and SSG2 (estimate = −5.875; mean difference ± SE = 5.875 ± 1.45; 95% CI = −8.73, −3.02; Cohen’s d = 1.32, large) and between SSG1 and SSG3 (estimate = −8.25; mean difference ± SE = 8.25 ± 1.45; 95% CI = −11.1, −5.4; Cohen’s d = 1.88, large). Also, the NB in the TSAP varied significantly depending on the type of SSG (F = 4.784; *p* = 0.016). The post hoc test (Bonferroni) revealed a significant difference between SSG1 and SSG2 (estimate = −2.813; mean difference ± SE = 2.813 ± 1.10; 95% CI = −4.97, −0.657; Cohen’s d = 0.72, moderate) and between SSG1 and SSG3 (estimate = −3.063; mean difference ± SE = 3.063 ± 1.1; 95% CI = −5.22, −0.907; Cohen’s d = 0.81, moderate).

Furthermore, results indicated that LB in the TSAP varied significantly according to the type of SSG (F = 3.646; *p* = 0.035). The post hoc test (Bonferroni) revealed a significant difference between SSG1 and SSG3 (estimate = −1.813; mean difference ± SE = 1.813 ± 0.7; 95% CI = −3.19, −0.44; Cohen’s d = 0.82, moderate). In particular, the LB parameter was significantly influenced by the interaction between SSG and age category (SSGCategory) (F = 6.512; *p* = 0.003). The post hoc test (Bonferroni) revealed a significant difference between the SSG1Category (estimate = −4.75; mean difference ± SE = 4.75 ± 1.401; 95% CI = −7.5, −2.004) and SSG2*Category (estimate = −3.875; mean difference ± SE = 3.875 ± 1.401; 95% CI = −6.62, −1.129).

The analysis suggests that the type of SSG significantly influenced OB values in the TSAP (F = 7.555; *p* = 0.002). The post hoc test (Bonferroni) revealed a significant difference between SSG1 and SSG2 (estimate = −2; mean difference ± SE = 2 ± 0.718; 95% CI = −3.41, −0.592; Cohen’s d = 0.84, moderate) and between SSG1 and SSG3 (estimate = −2.688; mean difference ± SE = 2.688 ± 0.718; 95% CI = −4.1, −1.28; Cohen’s d = 1.30, large). Furthermore, the type of SSG significantly influenced SS values in the TSAP (F = 7.168; *p* = 0.003). The post hoc test (Bonferroni) revealed a significant difference between SSG1 and SSG3 (estimate = −1.625; mean difference ± SE = 1.625 ± 0.431; 95% CI = −2.469, −0.781; Cohen’s d = 1.19, moderate).

The type of SSG produced significant effects on EI values in the TSAP (F = 6.599; *p* = 0.004). The post hoc test (Bonferroni) revealed a significant difference between SSG1 and SSG3 (estimate = −0.259; mean difference ± SE = 0.259 ± 0.072; 95% CI = −0.4, −0.117; Cohen’s d = 1.20, large).

Finally, the analysis showed that PS in the TSAP was significantly influenced by the type of SSG (F = 18.574; *p* < 0.001). The post hoc test (Bonferroni) revealed a significant difference between SSG1 and SSG2 (estimate = −4.63; mean difference ± SE = 4.63 ± 1.13; 95% CI = −6.84, −2.42; Cohen’s d = 1.35, large) and between SSG1 and SSG3 (estimate = −6.710; mean difference ± SE = 6.71 ± 1.13; 95% CI = −8.92, −4.5; Cohen’s d = 2.05, very large).

The Spearman’s correlation analysis between final RPE and TSAP technical-tactical indices did not show any significant associations for any of the variables (*p* > 0.05).

## 4. Discussion

This study aimed to investigate the effects of 4 vs. 4 SSGs played with three different playing areas (SSG1: 13 × 12 m; SSG2: 20 × 13 m; and SSG3: 25 × 17 m) on players’ technical and tactical performance, as well as subjective measures of internal load and enjoyment, among U16 and U18 elite youth water polo players.

The main findings suggest the following conclusions: (a) RPE values, and consequently s-RPE values, during the three game periods and at the end of the training session were influenced by the SSG format; (b) the individual values of perceived exertion exhibited a similar trend across the three game periods; (c) the enjoyment values showed differences between the two players’ age categories; (d) the Enjoyment and RPE values of the entire session showed no significant relationship; and (e) the TSAP analysis showed significant differences across all variables, as well as SSG format effects.

The SSG format, which included the use of only one goal (SSG1) or two goals (SSG2 and SSG3) and a different time available for first and second ball possession, influenced RPE and s-RPE values during the three playing periods and at the end of the training session. In particular, the SSG1 showed lower values compared to the other two formats, which is consistent with previous studies in team sports, indicating that the manipulation of playing tasks and playing area can influence individual perceived exertion [15,24,25] as well as performance variations during the playing periods [26]. In our case, this effect may be attributed to the differences in playing area and playing tasks (one vs. two goals and different time available for first and second possession) across the three SSG formats. Specifically, the SSG2 and SSG3, played with two goals and a larger playing area, required players to cover greater distances and manage more transitional situations due to the higher number of changes of defensive/attacking phases in relation to complex player positioning at the end of the transition phase, leading to higher physical demands and perceived exertion. In contrast, SSG1, played with one goal and the smallest area, may have limited high-intensity displacements while increasing the frequency of technical actions, resulting in lower overall perceptions of internal load.

Additionally, the structure of the tasks and the available ball time possession for decision-making could have influenced players’ pacing strategies, further contributing to the differences observed in RPE and s-RPE. The correlation results indicate that individual perceived exertion values exhibited a similar trend across the three periods of each SSG. In particular, players who reported higher levels of fatigue in the first period tended to maintain similar levels in the subsequent periods. These findings are consistent with previous studies, suggesting that perceived exertion in water polo does not vary randomly but follows a stable pattern throughout the match [26,27]. Moreover, the comparison between the mean RPE values for the three game periods and the final RPE can be considered a valid indicator of overall fatigue [28,29]. Unlike the other variables, the enjoyment values showed differences between age categories, with U16 players reporting higher enjoyment compared to U18 players. According to Clemente [30], younger athletes tend to experience the playful aspect of training with less pressure, whereas older players approach the game with greater maturity. Similar findings have also been reported in the youth water polo literature, highlighting the importance of providing continuous coaching support to stimulate the motivation of young players during training activities and prevent performance decline or excessive fatigue [31,32,33].

Furthermore, no significant relationship was found between enjoyment and final RPE. This result is consistent with Selmi et al. [29], suggesting that enjoyment is not directly dependent on players’ perceived exertion. Therefore, players can experience high levels of enjoyment even under high physical stress conditions. In this context, emotional factors may help players even in demanding playing situations. For this reason, it is essential for water polo coaches to design training sessions that are not only intense but also well-structured and engaging in order to maintain high levels of motivation among players. The TSAP analysis showed significant differences across all variables (RB, CB, VP, NB, LB, OB, SS, EI, and PS), with SSG2 and SSG3 presenting lower values compared to SSG1. As the playing area increases, the game conditions (two goals and distribution of players’ square meters of playing area) become more similar to real match situations. In these scenarios, players’ ability to manage ball possession and recognize scoring opportunities becomes more evident [34]. Among the TSAP variables observed in our study, only LB showed a significant interaction between SSG format and players’ age category, suggesting that the impact of this variable is not consistent across U16 and U18 groups.

Therefore, in youth water polo, the role of the coach becomes crucial in evaluating whether players are ready to perform under certain task constraints and if these SSGs can be used to stimulate improvements in specific aspects. Overall, TSAP values were higher in SSG1 compared to the other formats, indicating that players had more opportunities to make decisions and express creativity. Consequently, the format of SSG1, with one goal and less time available for first and second possession, appears to be a more effective game for improving technical-tactical skills in youth players. In contrast, SSG2 and SSG3 formats reduce the number of ball possession opportunities while making those opportunities more meaningful for the development of ball management skills. This difference highlights the importance of using different format of SSGs, with SSG1 increasing the quantity of actions, while SSG2 and SSG3 emphasize the quality of ball management. Futhermore, considering that no significant correlations were found between final RPE and TSAP technical-tactical indices (Volume of Play and Performance Score) a higher perception of fatigue does not appear to negatively affect technical-tactical performance or the quality of ball management, as previously suggested in the literature [12,29]. However, in youth water polo this may also depend on the age of the players and, above all, on their level of experience.

Regarding age category, no significant differences were found for either RPE or TSAP variables (except for LB). Therefore, in our study, the two-year age difference does not seem to be a discriminating factor for the observed variables. This finding is consistent with Clemente et al. [35], who reported that, in elite youth settings, increasing competitive level tends to reduce differences related to chronological age. In this context, the use of SSGs during the training week may represent an effective sport-specific training strategy, as previously highlighted [12,36], promoting the development of technical-tactical skills while simultaneously enhancing player engagement, specific swimming potential [37] and accessibility to training.

### Limitations

The study provides relevant practical implications but also presents some methodological limitations. The first limitation concerns the small sample size and the competitive level of the participants (elite U16 and U18). This sample selection suggests that the findings may not be generalizable to lower-level teams or different age categories, as differences in technical, physical, and experiential aspects may exist. In addition, the absence of elite senior players does not allow for a direct comparison across performance levels.

Another limitation relates to the assessment methods, which were based only on subjective measures (RPE and Enjoyment), without including objective physiological data. The use of more precise tools, such as heart rate monitors, could have provided more detailed information on players’ physiological responses.

Furthermore, the study was conducted over a limited period, specifically during the preparation phase for the final stages of the season. As a result, each SSG format was performed only once per category. Therefore, players’ responses may vary depending on the period of the season.

Future research should consider using these monitoring tools over longer periods to obtain a more comprehensive and dynamic understanding of both physical and technical-tactical performance.

## 5. Conclusions

In conclusion, this study shows that SSG format influences both perceived load and technical-tactical performance. Specifically, a smaller playing area, the use of one goal and shorter time available for ball possession significantly increases the frequency of actions, whereas a larger playing area, the use of two goals and longer time available for ball possession allows players to develop ball management skills under greater fatigue and promotes higher swimming demands, improving players’ swimming potential during game-based training.

These findings provide practical implications for water polo coaches and strength and conditioning practitioners, supporting the design and monitoring of targeted training sessions. Depending on the training objective, it is important to select and adapt the SSG format accordingly in order to balance physical load and technical-tactical development.

In addition, RPE and Enjoyment scales were confirmed as useful tools for coaches and technical staff to monitor both the physical and mental state of players.

Future studies should include a larger sample size and involve more teams, in order to better understand whether the types of training performed during the week can effectively translate into improved match performance.

### Practical Applications

The results of this study show that SSGs are effective tools to develop both physical and technical-tactical aspects in athletes. Water polo coaches may consider the following:

- Smaller playing areas during low-load training sessions reduce excessive perceived fatigue while maintaining active technical-tactical involvement;
- Larger playing areas can be applied to reproduce transition situations between attack and defense, better simulating match-related dynamics and swimming demands;
- The RPE scale represents a simple and practical tool for monitoring athletes’ perceived internal load during both training sessions and official matches;
- The Enjoyment scale can help to monitor players’ motivation and perceived fun, supporting the selection of more engaging training exercises;
- The TSAP can be used not only during official matches, but also during daily training sessions and friendly games to continuously monitor individual technical-tactical performance;
- Through TSAP analysis, players’ difficulty in ball management and decision-making during game situations can be identified, allowing more individualized training adjustments;
- The combination between SSG, RPE, Enjoyment, and TSAP provides a multidimensional overview of players’ physical, technical-tactical, and psychological responses to training.

## Figures and Tables

**Table 1:** TSAP components in water polo *.

GAINING POSSESSION OF THE BALL	
Receiving the ball (**RB**)	The player receives the ball from a partner and does not immediately lose control of it.
Conquering the ball (**CB**)	A player is considered having conquered the ball if he or she intercepted it, stole it from an opponent, or recaptured it after an unsuccessful shot on goal or after a near-loss to the other team.
**DISPOSING OF THE BALL**	
Playing a neutral ball (**NB**)	A routine pass to a partner or any pass which does not truly put the other team in jeopardy is considered a neutral ball.
Losing the ball (**LB**)	A player is considered having lost the ball when he or she loses it to the other team without having scored a goal (Shots, Passages, Lost Balls, Contrafouls).
Playing an offensive ball (**OB**)	An offensive ball is a pass to a partner which puts pressure on the other team and, most often, leads to a shot on goal (Assists, Offensive passages, or Center Balls) or a gained exclusion with the ball in the hand
Executing a successful shot (**SS**)	A shot is considered successful when it scores or possession of the ball is retained by one’s team (Goals and Shots)
**PERFORMANCE INDICATORS**	
Volume of Play (**VP**)	VP: RB + CB
Efficiency Index (**EI**)	EI: (OB + SS)/(10 + LB)
Performance Score (**PS**)	PS: (VP/2) + (EI × 10)

* Adapted from Grehaigne et al. [18].

**Table 2:** Mean ± SD of final RPE and Enjoyment values for the two categories across the different SSG formats.

SSG	Perception Scales	U16	U18
SSG1	RPE	6 ± 1.51	7.13 ± 0.83
	ENJ	5.88 ± 0.64	4.63 ± 0.74
SSG2	RPE	7.25 ± 1.28	7.75 ± 0.89
	ENJ	5.5 ± 0.53	5.25 ± 0.71
SSG3	RPE	7 ± 1.51	8 ± 0.76
	ENJ	6 ± 0.93	4.88 ± 0.64

Note. RPE = Rate of perceived exertion; ENJ = Enjoyment Likert scale.

**Table 3:** Total TSAP events across the different SSG formats for both categories.

SSG	TSAP Parameters	U16	U18	TOTAL
SSG1	RB	170	163	333
	CB	23	40	63
	VP	193	203	396
	NB	106	100	206
	LB	30	52	82
	OB	41	39	80
	SS	29	26	55
	EI	5.07	4.16	9.23
	PS	147.15	143.08	290.23
SSG2	RB	146	131	277
	CB	14	11	25
	VP	160	142	302
	NB	80	81	161
	LB	38	22	60
	OB	29	19	48
	SS	16	24	40
	EI	3.17	3.35	6.52
	PS	111.66	104.49	216.15
SSG3	RB	115	119	234
	CB	18	12	30
	VP	133	131	264
	NB	75	82	157
	LB	31	22	53
	OB	22	15	37
	SS	13	16	29
	EI	2.55	2.53	5.08
	PS	92.05	90.82	182.87

Note. SSG—small-sided game; RB—receiving the ball; CB—conquering the ball; VP—volume of Play; NB—playing a neutral ball; LB—losing the ball; OB—playing an offensive ball; SS—executing a successful shot; EI—Efficiency Index; PS—performance score.

**Table 4:** Mean ± SD of TSAP variables for the two categories across the different SSG formats.

SSG	TSAP Parameters	U16	U18
		Mean ± SD	Mean ± SD
SSG1	RB	21.25 ± 4.68	20.38 ± 6
	CB	2.88 ± 3.23	5 ± 1.51
	VP	24.13 ± 3.76	25.38 ± 5.53
	NB	13.25 ± 4.5	12.5 ± 3.74
	LB	3.75 ± 0.89	6.5 ± 2.83
	OB	5.13 ± 2.3	4.88 ± 3.09
	SS	3.63 ± 1.51	3.25 ± 1.39
	EI	0.63 ± 0.23	0.52 ± 0.32
	PS	18.39 ± 3.37	17.88 ± 4.49
SSG2	RB	18.25 ± 5.01	16.38 ± 3.74
	CB	1.75 ± 1.04	1.38 ± 0.92
	VP	20 ± 4.54	17.75 ± 3.99
	NB	10 ± 3.82	10.13 ± 3.98
	LB	4.75 ± 2.31	2.75 ± 1.49
	OB	3.63 ± 2	2.38 ± 2.13
	SS	2 ± 0.93	3 ± 1.2
	EI	0.41 ± 0.18	0.42 ± 0.2
	PS	13.73 ± 3.38	13.06 ± 2.81
SSG3	RB	14.38 ± 4.14	14.88 ± 3.56
	CB	2.25 ± 1.91	1.5 ± 1.41
	VP	16.63 ± 5.01	16.38 ± 3.54
	NB	9.38 ± 3.78	10.25 ± 3.58
	LB	3.88 ± 2.03	2.75 ± 1.75
	OB	2.75 ± 1.04	1.88 ± 1.46
	SS	1.63 ± 0.92	2 ± 1.69
	EI	0.32 ± 0.07	0.32 ± 0.16
	PS	11.51 ± 2.84	11.35 ± 2.48

Note. SSG—small-sided game; RB—receiving the ball; CB—conquering the ball; VP—volume of Play; NB—playing a neutral ball; LB—losing the ball; OB—playing an offensive ball; SS—executing a successful shot; EI—Efficiency Index; PS—performance score.

**Table 5:** Spearman’s correlation (rho and *p*-value) between final RPE and TSAP variables and indices.

FINAL RPE	RB	CB	VP	NB	LB	OB	SS	EI	PS
Spearman’s rho	−0.22	0.01	−0.25	−0.04	−0.1	−0.16	−0.2	−0.16	−0.23
*p*-value	0.13	0.93	0.09	0.8	0.51	0.28	0.18	0.29	0.12

Note. RB—receiving the ball; CB—conquering the ball; VP—volume of Play; NB—playing a neutral ball; LB—losing the ball; OB—playing an offensive ball; SS—executing a successful shot; EI—Efficiency Index; PS—performance score.

## Data Availability

Data are contained within the article.

## Abbreviations

The following abbreviations are used in this manuscript:

RPE	Rated Perceived Exertion
s-RPE	Session Rated Perceived Exertion method
TSAP	Team Sport Assessment Procedure
RB	Receiving the ball
CB	Conquering the ball
NB	Playing a neutral ball
LB	Losing the ball
OB	Playing an offensive ball
SS	Executing a successful shot
VP	Volume of Play
EI	Efficiency Index
PS	Performance score
